# The Functional Tactile Object Recognition Test: A Unidimensional Measure With Excellent Internal Consistency for Haptic Sensing of Real Objects After Stroke

**DOI:** 10.3389/fnins.2020.542590

**Published:** 2020-09-23

**Authors:** Leeanne M. Carey, Yvonne Y. K. Mak-Yuen, Thomas A. Matyas

**Affiliations:** ^1^Department of Occupational Therapy, Social Work and Social Policy, School of Allied Health, Human Services and Sport, College of Science, Health and Engineering, La Trobe University, Melbourne, VIC, Australia; ^2^Neurorehabilitation and Recovery, The Florey Institute of Neuroscience and Mental Health, Heidelberg, VIC, Australia

**Keywords:** somatosensation, haptic, object recognition, perception, touch, stroke, assess, sensation

## Abstract

**Introduction:**

Our hands, with their exquisite sensors, work in concert with our sensing brain to extract sensory attributes of objects as we engage in daily activities. One in two people with stroke experience impaired body sensation, with negative impact on hand use and return to previous valued activities. Valid, quantitative tools are critical to measure somatosensory impairment after stroke. The functional Tactile Object Recognition Test (fTORT) is a quantitative measure of tactile (haptic) object recognition designed to test one’s ability to recognize everyday objects across seven sensory attributes using 14 object sets. However, to date, knowledge of the nature of object recognition errors is limited, and the internal consistency of performance across item scores and dimensionality of the measure have not been established.

**Objectives:**

To describe the original development and construction of the test, characterize the distribution and nature of performance errors after stroke, and to evaluate the internal consistency of item scores and dimensionality of the fTORT.

**Method:**

Data from existing cohorts of stroke survivors (*n* = 115) who were assessed on the fTORT quantitative measure of sensory performance were extracted and pooled. Item and scale analyses were conducted on the raw item data. The distribution and type of errors were characterized.

**Results:**

The 14 item sets of the fTORT form a well-behaved unidimensional scale and demonstrate excellent internal consistency (Cronbach alpha of 0.93). Deletion of any item failed to improve the Cronbach score. Most items displayed a bimodal score distribution, with function and attribute errors (score 0) or correct response (score 3) being most common. A smaller proportion of one- or two-attribute errors occurred. The total score range differentiated performance over a wide range of object recognition impairment.

**Conclusion:**

Unidimensional scale and similar factor loadings across all items support simple addition of the 14 item scores on the fTORT. Therapists can use the fTORT to quantify impaired tactile object recognition in people with stroke based on the current set of items. New insights on the nature of haptic object recognition impairment after stroke are revealed.

## Introduction

Our hands, with their exquisite sensors, work in concert with our sensing brain to extract sensory attributes of objects to interact with those objects as we engage in our daily activities. This ability is critical to tactually recognize objects (e.g., a cup from a jar), locate objects (e.g., locate a button from the background of the clothing on which it is fastened), appreciate the tactile features of objects (e.g., the shape and warmth of a child’s hand), and to connect with the people and objects that we interact with in the immediate (reachable) space around us.

The capacity underlying these tasks is commonly referred to as tactile (or haptic) object recognition. Tactile (haptic) object recognition is the ability to identify common objects through the use of touch without the aid of vision. Haptic object recognition relies on all the somatosensory inputs used by the tactile system and skin sensors in combination with information from position and movement sensors in joints and muscles and force receptors in tendons (Lederman and Klatzky, 1990, 2009). It involves extraction of various object attributes and the integration of that information to recognize what the object is. The sensory object attributes extracted include texture, shape, size, weight, temperature, hardness, and function/motion of objects (Lederman and Klatzky, 1987, 1990). Haptic perception typically involves active manual exploration. When people use their haptic system, they typically focus on their experiences of the external world and objects and their properties, such as roughness, shape, and weight (Lederman and Klatzky, 2009).

One in four adults are likely to suffer a stroke, based on the estimated global lifetime risk of stroke (Feigin et al., 2018). One in two stroke survivors experience impairment in the ability to receive and interpret body sensations such as touch, limb position sense, and to recognize objects through touch (Carey, 1995; Connell et al., 2008; Tyson et al., 2008; Carey and Matyas, 2011; Kessner et al., 2016). It is like the hand is blind (Turville et al., 2019). The person has difficulty holding and using simple objects such as a fork, and frequently learns not to use his/her hand. The impairment negatively impacts the person’s ability to interact with the world around them (Connell et al., 2014; Turville et al., 2019), hand function (Blennerhassett et al., 2007, 2008), goal-directed use of the arm (Jeannerod, 1997; Turville et al., 2017), and return to previous life activities (Carey et al., 2016b, 2018). It is associated with poorer functional outcome (Reding and Potes, 1988; Carey et al., 2016b), yet it is a “neglected” area of stroke rehabilitation (Kalra, 2010). Valid, quantitative measurement is critical to diagnose somatosensory impairment and assess change over time (Carey, 1995).

Assessment of the ability to recognize common objects through the sense of touch is important after stroke. It has face validity for the person with stroke and allows direct translation of capacity to the context of everyday tasks. Some measures have been developed to assess recognition of a subset of object features such as shape and size, often using a two-dimensional layout (Rosen and Lundborg, 1998) or arbitrary shapes (Kalisch et al., 2012). However, in the real world, we typically need to interact with three-dimensional (3D) common objects that have multiple sensory object features. Further, we know that real 3D common objects can be recognized very efficiently in non-neurologically impaired adults (Lederman and Klatzky, 1987). Haptic recognition of everyday objects is quite fast and highly accurate with 96% correctly named: 68% in less than 3 s and 94% within 5 s (Klatzky et al., 1985). Further, in using common objects, it may be important to not only recognize sensory features but also recognize the type of object, such as a drinking vessel (typically characterized by a cluster of object features).

Our overall objective was to develop a quantitative and psychometrically sound tool to measure the capacity of haptic object recognition using 3D common objects. Our approach involved two sub-aims:

1. To construct a quantitative measure of the ability to recognize everyday objects through touch, the functional Tactile Object Recognition Test (Part 1).
2. To evaluate the internal consistency of item scores and dimensionality of the functional Tactile Object Recognition Test, an evidence-based assessment to measure somatosensory impairment in the hand after stroke (Part 2).

## Part 1: Development and Construction of the Functional Tactile Object Recognition Test (fTORT)

The functional Tactile Object Recognition Test (fTORT) was developed to quantitatively measure tactile (haptic) object recognition in adult persons who experience stroke (Carey et al., 2006). The test has been designed to include common objects to maximize face validity and because humans are accurate and efficient at recognizing real 3D common objects by touch (Klatzky et al., 1985). The measure is designed to capture the interface between tactile exploration and sensing and to systematically sample haptic object recognition across a range of somatosensory attributes. This is the first full description of the development and construction of the fTORT by the originator of the tool.

### Selection of Test Items

In developing the assessment tool, it was first important to select objects that could be used to sample different attributes of somatosensation in the context of everyday objects. Seven sensory attributes of objects have been identified by Lederman and Klatzky (1987, 1990, 1993) based on the optimal exploratory procedures used to extract those sensory attributes. Lederman and Klatzky (1987) first systematically characterized the association between attributes of objects, such as shape and texture, and the movements (exploratory procedures) used to recognize those features. They used cluster analysis of the exploratory movements to classify the associated object attribute (Lederman and Klatzky, 1990). They then investigated the most optimal movements used to recognize 100 real 3D common objects across different functional categories important for knowledge-driven exploration (Lederman and Klatzky, 1990). The seven object attributes (e.g., shape) and the corresponding optimal exploratory procedure used to extract the attribute (e.g., contour following) are as follows: exact shape – contour following; volume/global shape – enclosure; texture – lateral motion; hardness – pressure; weight – unsupported holding; temperature – static contact; part motion or motion of a part – characteristic movement specific to the object (e.g., flick of a light switch) (Lederman and Klatzky, 1987, 1990).

Objects used for the current assessment were selected to represent the seven sensory object attributes, and corresponding optimal exploratory procedure used to recognize that attribute, as defined by Lederman and Klatzky (1987), and were selected from the set of 100 common objects described by them (Lederman and Klatzky, 1990). To capture a range of objects commonly encountered, the objects included were selected across the different object categories investigated, including household, personal, office, leisure, and food, and spanned large, medium, and small objects that were capable of being readily manipulated. Objects were selected to sample each of the seven sensory attributes twice. Thus, the test comprises 14 object sets. Object sets were also constructed to permit discrimination of the distinctive somatosensory attribute associated with that object set, by varying the specific sensory attribute (e.g., weight, shape) between object pairs. For example, in selecting objects to test temperature, a review of the 100 common objects revealed that objects such as metal doorknob (function category: door opener), wooden bowl (function category: container), and plastic paperclip (function category: paper fastener) were most optimally recognized via the sensory attribute of temperature, based on the matched exploratory procedure of static contact. In constructing the object sets for the fTORT, we selected doorknobs and bowls for object sets, with objects included having different surface temperatures, e.g., wooden and metal doorknob. Somatosensory attributes tested and the corresponding object sets are listed in Table 1.

**TABLE 1:** Somatosensory attribute sampled and corresponding test objects and object function category.

Sensory attribute tested	Test object	Matched pair	Object function category
*Weight*	Full milk bottle	Half-full milk bottle	Drink container
*Weight*	Empty jar	Full jar	Food jar
*Temperature*	Metal doorknob	Wooden doorknob	Door opener
*Temperature*	Stainless steel bowl	Plastic bowl	Container – bowl
*Hardness*	Hardcover book	Soft cover book	Reading material
*Hardness*	Firm plastic cup	Crushable plastic cup	Drinking vessel
*Function/motion*	Zipper	Buttons	Clothing fastener
*Function/motion*	Click switch	Turn switch	Wall attachment
*Shape (exact)*	Spoon	Fork	Eating utensil
*Shape (exact)*	Cylindrical pasta	Spiral-shaped pasta	Food
*Size (volume)*	Small faced watch	Large-faced watch	Timepiece
*Size (volume)*	House key	Filling cabinet key	Security device
*Texture*	Plastic card	Paper card	Office supplies
*Texture*	Wooden clothes peg	Plastic clothes peg	Clothes hanging device

### Object Sets and Response Poster

Object sets were constructed where two objects differed in the sensory attribute of interest (e.g., weight) whereas the third object in the set was a distractor object, i.e., that varied in the object attribute of interest but also in another attribute (e.g., weight and shape). Object sets had a common function, e.g., food jar, drinking vessel, and security device. These categories of function were based on the work of Lederman and Klatzky (1990).

**FIGURE 1:**
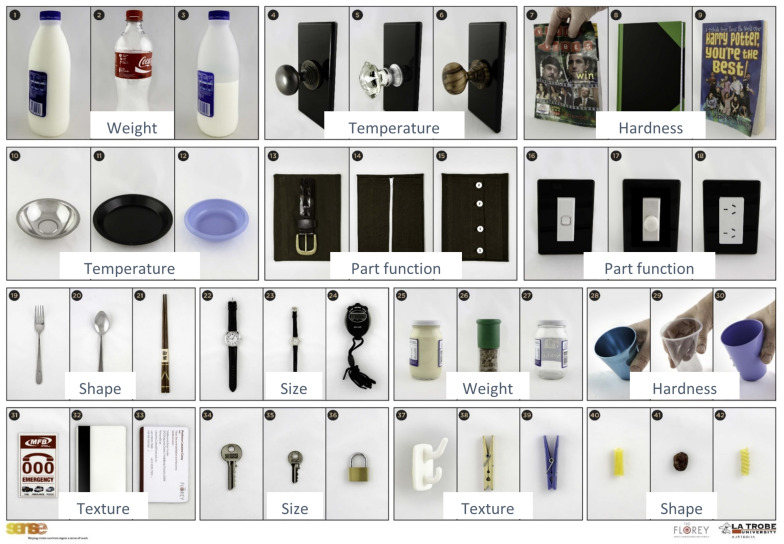
Functional Tactile Object Recognition Test response poster displaying object sets. Sensory attribute tested within object sets, e.g., weight, is labeled for each set in the figure. Figure adapted from Turville et al. (2018). Reproduced with permission. The final publication is available at IOS Press through http://dx.doi.org/10.3233/NRE-182439.

Each object set, 14 in total, was displayed visually on a photo response poster (see Figure 1). A response poster was used to restrict the number of possible responses and to facilitate ease of response for participants. Visual display of objects was selected given the face validity of this approach and the alignment of visual and tactile modalities when recognizing object properties, e.g., the shape of an object can be seen, and that visual image aligns with the tactile shape when explored haptically using contour following or enclosure (Lacey and Sathian, 2014). Use of a visual response poster that was in full view during object exploration also minimized memory-related demands. Each object (test item) may be described according to two main features:

1. Type of object and the object function category (object set) it belongs to, e.g., cup – drinking vessel; key – security device.
2. Sensory attribute it tests, e.g., weight – full jar/empty jar; hardness – crushable plastic cup/firm plastic cup.

For example, in the object set of bottles that have the same function (drink container), objects 1 and 3 are a matching pair that vary by weight only (i.e., one is a full milk bottle and the other a half-full milk bottle), whereas object 2, the distractor object, varies by weight (empty) but also by shape of the bottle (i.e., a Coke bottle) (see Figure 1).

### Test Scale and Scoring

Test scores were constructed to achieve a ranking in the type and amount of response error while sampling the seven object attributes. Response error for each object set permitted sampling whether the person could recognize the type of object through touch (i.e., object category of function, such as drinking vessel), the presence of the distinctive object attribute being tested (i.e., was it recognized relative to similar object types with distractor attributes, e.g., crushability of cup?), and the accuracy of attribute recognition (i.e., was the amount of distinctive sensory object attribute correctly identified, e.g., hardness of cup being firm or crushable?). Scoring according to these levels of recognition was operationalized according to the criterion descriptors outlined in Table 2. Each of the seven object attributes to be tested were sampled twice (i.e., use of two different object sets for a specific object attribute such as weight) and scored.

**TABLE 2:** Item response scoring according to the object and sensory attribute descriptors, with example.

Item Score	Response	Object match/error descriptor	Detailed description	Example: Test object is the half-full milk bottle (response given)
3	Correct object	Exact match	Object matched to correct category of function and correct amount of sensory attribute	Half-full milk bottle

2	Object pair	Error in distinctive sensory object attribute	Error in recognition/discrimination of amount of distinctive sensory attribute being tested. Object category correct	Full milk bottle (error in weight of bottle)

1	Object distractor	Error in two or more sensory object attributes	Error in recognition of the attribute being tested (weight) and at least one other attribute as evident in distractor object. Object category correct	Empty coke bottle (error in weight and shape of bottle)

0	Incorrect	Error of object type/function and sensory object attribute	A gross error of object type/function and sensory attribute. This error is more severe than being incorrect in even two sensory attributes	For example, food jar or reading material (different type of object category)

Item responses were scored according to descriptors in Table 2. This permitted quantification of the amount of error (using ordinal scale) within object sets. However, it is unclear whether these item error scores can be summed to give an overall error score for the fTORT. We therefore sought to examine empirically whether the item scores form a unidimensional scale permitting addition of item scores into a single total score.

## Part 2: Evaluation of the Internal Consistency of Item Scores and Dimensionality of the fTORT

The fTORT has been constructed, as detailed earlier, as a research and clinical tool to quantitatively measure tactile (haptic) object recognition using real 3D common objects. It has been used in clinical research settings to measure somatosensory impairment within several studies. Preliminary findings indicate that the tool has good discriminative validity to detect impairment in people with stroke relative to age-matched healthy controls (Carey et al., 2006). The purpose of the current empirical study was to establish the internal consistency of performance across item scores, and the dimensionality of the measurement scale, e.g., whether haptic object recognition as tested using the fTORT can be represented on a single scale or not.

## Materials and Methods

### Sample: Participants, Study Cohorts, and Study Design

Baseline data from existing cohorts of stroke survivors who were assessed on the fTORT were extracted and pooled. This included data from 115 stroke survivors who were enrolled in the following studies: SENSe (*S*tudy of the *E*ffectiveness of *N*eurorehabilitation on *Se*nsation; *n* = 52) (Carey et al., 2011), CoNNECT (*Co*nnecting *N*ew *N*etworks for *E*veryday *C*ontact through *T*ouch; *n* = 45) (Carey, 2013; Goodin et al., 2018), and IN_Touch (*I*maging *N*europlasticity of *Touch*; *n* = 18) (Bannister et al., 2015; Carey et al., 2016a). There were no overlapping participants across studies.

Data were extracted and pooled across these existing cohorts of stroke survivors who had similar characteristics and inclusion/exclusion criteria. Stroke participants were medically stable, and able to give informed consent and comprehend simple instructions. Exclusion criteria included evidence of unilateral spatial neglect based on standard neuropsychological testing, previous history of other central nervous system dysfunction, or peripheral neuropathy. Additional selection criteria for the CoNNECT and IN_Touch studies included participants being right-handed dominant, no brainstem infarct, first episode infarct, and being suitable for MRI. All participants gave voluntary informed consent and procedures were approved by Human Ethics committees of participating hospitals and La Trobe University, Australia.

All participants were assessed at baseline on the fTORT. Timing of the baseline assessment post-stroke varied across the studies, from a median of 4 weeks to 53 weeks post-stroke. The fTORT was administered to assess tactile object recognition both for the hand contralateral to the side of lesion (“affected” hand) and ipsilateral to the lesion (commonly referred to as the “unaffected” hand). Data included in the current study relate to scores for the “affected” hand contralateral to the side of lesion only.

### Measure: fTORT

The fTORT is designed to test recognition of objects through the sense of touch. Test equipment includes 14 actual test objects to be felt and 14 matched pair objects (Table 1); response poster displaying 14 object sets, i.e., 42 objects in total (Figure 1); five display objects – for size calibration (metal bowl, desert spoon, full jar, paper business card, and house key); trial object (Coke bottle); curtain to occlude vision; mat to minimize any sound if object is dropped; ear muffs to minimize identification of object via sound made when exploring the object; stop watch; waist height table; two chairs; and assessment form and pen.

#### Set-Up

The therapist sits opposite or to the side of the person being tested, depending on which arm is being tested (i.e., if the right arm is to be tested, sit on the right side of the person). A screen is placed in front of or to the side of the person to occlude vision of the test object. The poster of the test and distractor objects is placed on the table at a comfortable viewing distance. Objects used for size calibration are positioned along the top of the poster, in the same orientation as the object in the poster. The person’s hand to be tested is placed through the screen with their palm facing up and their arm resting on the table. Posture variations are allowed if required due to positioning restrictions or motor impairment, e.g., unable to achieve supination position due to tonal changes. A padded mat is placed under the test arm to minimize noise if the object is dropped. The person is instructed to put on the ear muffs to minimize any auditory clues from the test items. A stopwatch, test form, and pen are nearby for testing. During testing, the actual test objects are kept out of the person’s view.

#### Testing Procedure

**FIGURE 2:**
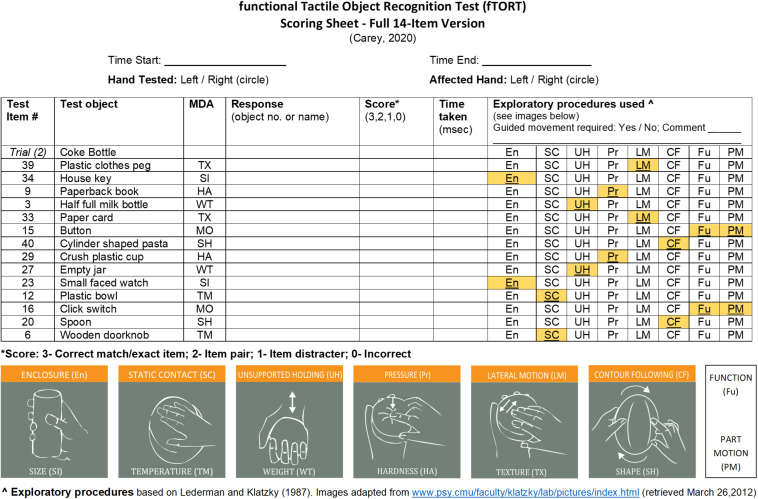
Assessment form for the functional Tactile Object Recognition Test (fTORT). MDA, most diagnostic attribute, i.e., the sensory attribute that distinguishes the test object from the object pair for each of the object sets; TX, texture; SI, size; HA, hardness; WT, weight; MO, motion; SH, shape; TM, temperature.

During each trial, one object from each object set (14 in total) is presented to the person using standard test instructions. The test items are listed on the assessment form (Figure 2). The test item (object) is placed in the person’s hand to be tested or the person’s hand is placed on the object, behind the curtain, in a standard manner. Only one hand, the tested hand, is allowed to be used to explore the object. The person is told that it is important to select the object that most closely matches what he/she feels from the response poster (comprising 42 everyday objects or 14 item sets), that not all objects will be used, and the same object may be presented on more than one occasion. The participant may need to be encouraged to look at all the object photographs before choosing their final answer. The person is instructed that as soon as he/she recognizes which object it is from the 14 object sets shown in the poster, they should put the object down and indicate the matching object by either pointing to the object or saying the identifying number of that object, for example, “27” (empty jar). They are instructed not to feel the object any more once they have given their response. The time to identification is recorded in milliseconds. The exploratory procedures (EPs) used by the person are also recorded. The assessor circles the EPs observed. The EP that is most optimal for the object pair is highlighted on the assessor sheet. People with stroke may need assistance to adequately explore the object. In this instance, the assessor helps the person explore the object using the most optimal exploratory procedure, as highlighted for that object set, in a standard manner. Thus the “standard” manner is matched to the object set and the guidance required (either moving the participant’s hand or moving the object) is provided in a way that simulates the optimal exploratory procedure for that object set. For example, if the set relates to weight, then the assessor would assist the person to achieve the unsupported holding exploratory procedure. Level of assistance required is recorded on the assessment form. Four different test protocol versions were available for testing.

### Data Analysis

Test scores were extracted and pooled. Four protocol versions were employed that varied in the order in which item sets were presented and/or which object in the matched pair was presented. After appropriate alignment of the item scores across the four protocol versions, a complete sample of test scores for 115 participants, each with 14 item scores, was available for analysis. Item and scale analyses were conducted on the raw item data. Distributions of item and total scores were determined and displayed graphically. Internal consistency of item scores was quantified using Cronbach alpha. Dimensionality analysis was conducted using principal component analysis.

## Results

### Background Characteristics of the Sample

Background data on age, sex, side of lesion, and time post-stroke for participants are presented in Table 3.

**TABLE 3:** Demographic and clinical characteristics of pooled stroke sample.

Demographics	Pooled sample (*n* = 115)	SENSe cohort (*n* = 52)	CoNNECT cohort (*n* = 45)	IN_Touch cohort (*n* = 18)
Age, *years, M (SD)*	58 (14)	61 (13)	53 (14)	60 (15)
Gender, *n (%)* Men Women	81 (70) 34 (30)	38 (73) 14 (27)	32 (71) 13 (29)	11 (61) 7 (39)
Lesion type, *n (%)* Cortical Subcortical Both Unknown	49 (43) 42 (36) 16 (14) 8 (7)	15 (29) 17 (33) 12 (23) 8 (15)	26 (58) 15 (33) 4 (9) 0 (0)	8 (44) 10 (56) 0 (0) 0 (0)
Stroke type, *n (%)* Ischemic Hemorrhagic	84 (73) 31 (27)	34 (65) 18 (35)	32 (71) 13 (29)	18 (100) 0 (0)
Hemisphere affected, *n (%)* Right Left Both	48 (42) 65 (56) 2 (2)	31 (60) 21 (40) 0 (0)	21 (47) 22 (49) 2 (4)	12 (67) 6 (33) 0 (0)
Handedness, *n (%)* Right Left	110 (96) 5 (4)	47 (90) 5 (10)	45 (100) 0 (0)	18 (100) 0 (0)
Affected side, *n (%)* Dominant Non-dominant	64 (56) 51 (44)	30 (58) 22 (42)	22 (49) 23 (51)	12 (67) 6 (33)
Time post-stroke, *weeks, median (IQR)*	40 (15–78)	45 (21–129)	53 (30–81)	4 (3–6)
Level and frequency of physical assistance provided^*a*^, *n (%)* Fully guided Partial guided No guidance Uncertain	*for n* = *97* 37 (38) 17 (17) 16 (16) 27 (28)	24 (46) 3 (6) 4 (8) 21 (40)	13 (29) 14 (31) 12 (27) 6 (13)	N/A N/A N/A N/A
Motor activity log^*b*^, *Average score/item*, *Median (IQR) Range*	2.1 (0.6–3.5) 0–4.94	1.3 (0.2–2.4) 0–4.93	2.6 (1.6–3.6) 0–4.42	2.8 (0.9–4.7) 0–4.94

*SENSe, Study of the Effectiveness of Neurorehabilitation on Sensation; CoNNECT, Connecting New Networks for Everyday Contact Through Touch; IN_Touch, Imaging Neuroplasticity of Touch; M, mean; SD, standard deviation; IQR, interquartile range; N/A, not available. ^*a*^Level of physical assistance from blinded assessors during testing using the functional Tactile Object Recognition Test. Presence and type of guidance was summarized from assessor comments by authors (YM-Y and LC). If assessors did not specify presence or level of guidance, the data for those participants were categorized as “Uncertain.” ^*b*^Motor activity log (MAL) – average score per item (total/number of valid items) to measure perceived “Amount of Use” of the arm in daily activities. Please note: SENSe and IN_Touch used the 30-item version and CoNNECT used the 14-item version of the MAL.*

### Distributions of Item Scores and Relationship to Total Scores

#### Distributions of Item Scores

**FIGURE 3:**
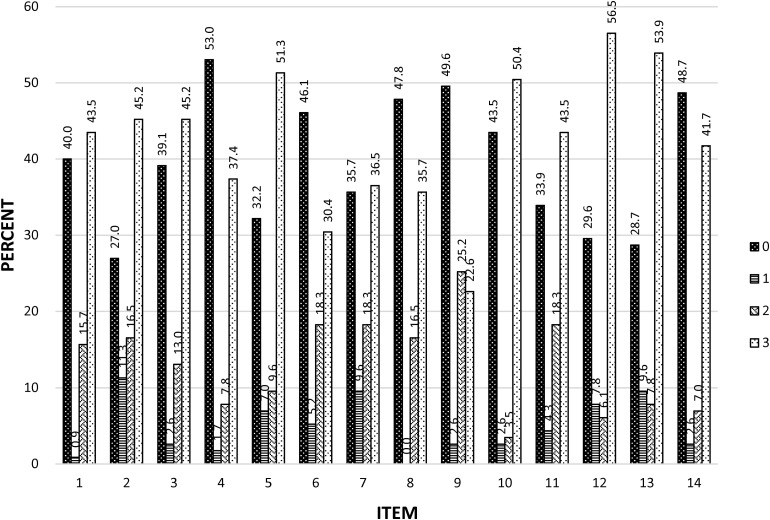
Distributions of fTORT item scores. Distribution of 0, 1, 2, and 3 scores for each of the 14 test items. Values above each bar are the percent of cases showing each score out of the total 115 independent scores available for each item. The sensory attribute tested and corresponding test object pair for each test item set are as follows: item 1 = shape (spoon/fork); item 2 = temperature (metal/wooden doorknob); item 3 = temperature (stainless steel/plastic bowl); item 4 = texture (paper/plastic card); item 5 = function/motion (zipper/buttons); item 6 = size (small-faced/large-faced watch); item 7 = weight (full/empty jar); item 8 = size (house key/filing cabinet key); item 9 = texture (wooden/plastic clothes peg); item 10 = hardness (hardcover book/soft cover book); item 11 = weight (full/half-full milk bottle); item 12 = hardness (firm/crushable plastic cup); item 13 = function/motion (click switch/turn switch); item 14 = shape (cylindrical pasta/spiral shaped pasta).

The distributions of scores for each item set of the fTORT are presented in Figure 3 for the sample of 115 participants (scaled in percentages out of the 115 cases). Most items, with only one exception (item 9), displayed a bimodal score distribution, with pronounced modes at scores of 0 and 3, i.e., errors of object function and sensory attribute (score 0), or exact match including sensory attribute (score 3). Only a minority of cases demonstrated errors solely in sensory attributes (i.e., scores of 1 or 2). For all items, except for item 9 (Wooden/Plastic Clothes Peg), markedly more participants committed object function and attribute errors (scoring 0) than either single or double sensory attribute errors (scores of 1 or 2). Two sensory attribute errors had a frequency from zero (item 8) to 11.3% (item 2) per item set. Single sensory attribute errors tended to be more frequent than two-attribute errors and ranged from a low of 3.5% (item 10) to a high of 25.2% for item 9. Item 9 was also the only item where a score of 2 was more common than a score of 3 and only by a small margin. The lowest mean score for a particular item set (possible range 0–3) was 1.2 for item 9 whereas the highest was 1.9 for item 12. Similar distributions of item scores are evident and item SDs are homogeneous, ranging from 1.27 to 1.44. All 14 item sets demonstrated that people can both correctly recognize or fail to recognize the test item; thus, none were either too easy or too difficult.

#### Relationship Between Frequency of Item Scores and Total Scores

**FIGURE 4:**
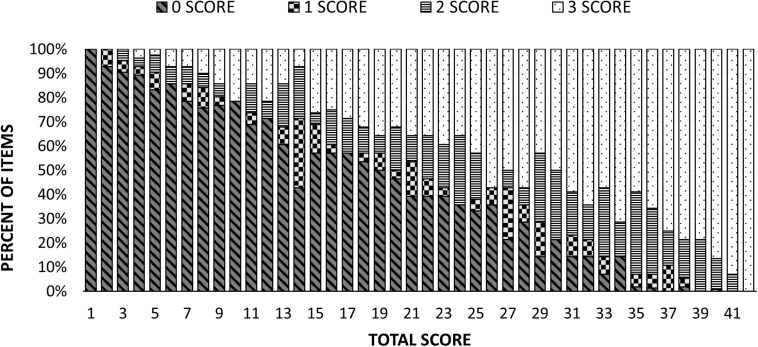
Cumulative bar plot showing pooled frequency of 0, 1, 2, and 3 item scores as a function of total score.

The cumulative bar plot (Figure 4) illustrates the proportion of items scoring 0, 1, 2, or 3 at each total score (sum over 14 items), obtained by pooling over cases with the same total score. As expected, the proportion of 0 scores diminished when total scores increased, whereas the proportion of 3 (completely correct) scores climbed at similar rates, a complementary inverse pattern. The proportion of errors restricted to specific sensory attributes, represented by scores of 1 and 2, were typically lower than object function and attribute errors (scores of 0) for most total score values. Specific somatosensory attribute errors consistently exceeded object function and attribute errors only for scores above 29.

#### Probability of Zero Scores

**FIGURE 5:**
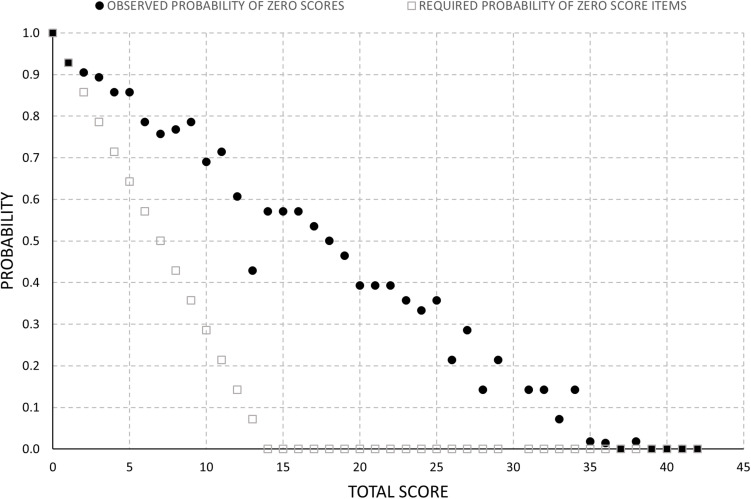
Observed probability of zero scores relative to the minimum probability required to achieve a given total score.

Given the very high proportion of zero scores, we investigated if this was greater than mathematically necessary. Scores as low as 14 can occur without a single item being a zero score, 13 with only item being a zero score, 12 if two items are allowed to be zero, etc. However, the probability of occurrence of zero score items observed in individuals at the same total score climbs steadily for scores below 35 (Figure 5), radically departing from the mathematically required probability, which is zero until 14 and only then climbs linearly. The difference between the observed probability of zero scoring items and that required to obtain each total score was statistically significant according to a Kolmogorov–Smirnov test (*p* < 0.001).

### Distribution of Total Scores, Internal Consistency of Items, and Dimensionality Analysis

#### Distribution of Total Scores

**FIGURE 6:**
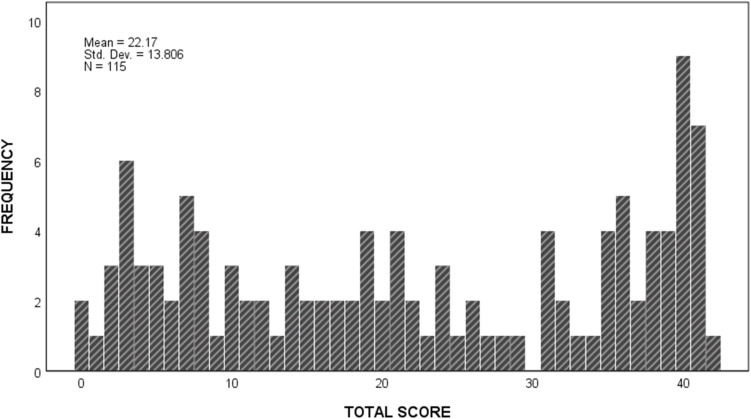
Frequency distribution of total fTORT scores.

Total scores were widely dispersed and displayed a relatively uniform distribution (Figure 6), ranging from the lowest to the highest possible scores, with an apparent slight increase in frequency at scores of 40 and 41. The total score distribution did not show a ceiling or floor effect.

#### Internal Consistency

Inter-item correlations ranged from 0.37 to 0.66. Cronbach’s alpha for the 14-item scale was 0.93, indicating very good internal consistency. Examination of item-total statistics (Table 4) showed that no improvement in internal consistency could be gained by deleting any of the 14 items. Variations in both scale mean and variance were comparable regardless of which item was deleted. Thus, coefficient alpha, item mean and item variance statistics do not offer a case for deletion of any of the 14 items.

**TABLE 4:** Item-total statistics and loadings on first principal component.

Item set	Scale mean if item deleted	Scale variance if item deleted	Corrected item-total correlation	Squared multiple correlation	Cronbach’s alpha if item deleted	Loadings on first component
Item 1	20.54	164.058	0.694	0.547	0.925	0.743
Item 2	20.37	167.901	0.640	0.522	0.926	0.695
Item 3	20.52	163.743	0.701	0.571	0.924	0.751
Item 4	20.87	162.921	0.705	0.552	0.924	0.756
Item 5	20.37	167.023	0.619	0.408	0.927	0.673
Item 6	20.83	163.894	0.733	0.597	0.923	0.781
Item 7	20.61	164.521	0.728	0.589	0.924	0.776
Item 8	20.77	163.339	0.714	0.601	0.924	0.764
Item 9	20.96	165.656	0.712	0.598	0.924	0.762
Item 10	20.56	164.267	0.646	0.462	0.926	0.701
Item 11	20.45	164.899	0.701	0.577	0.924	0.752
Item 12	20.27	166.725	0.631	0.510	0.927	0.686
Item 13	20.30	169.193	0.566	0.372	0.929	0.622
Item 14	20.75	165.173	0.632	0.460	0.927	0.685

*Total scale (14 items) statistics: mean = 22.17; variance = 190.6; Cronbach alpha = 0.930.*

#### Dimensionality

**FIGURE 7:**
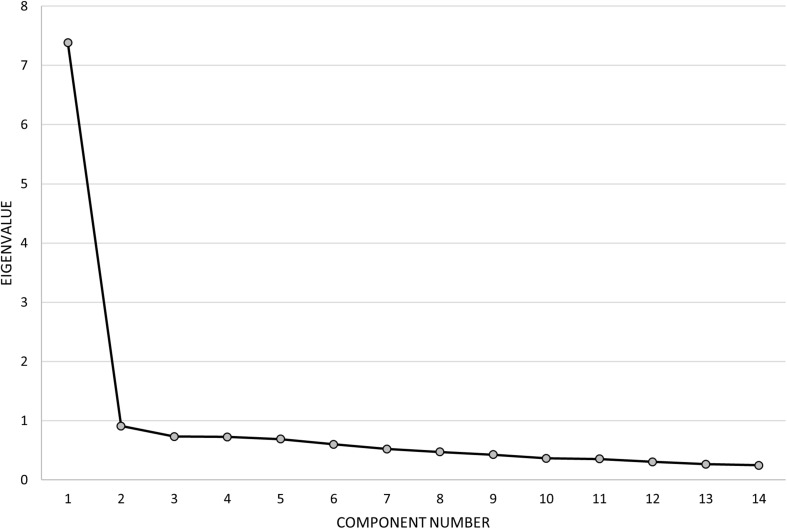
Scree plot summarizing eigenvalues obtained from the principal component analysis. A clear one-component solution is supported by the rapid drop in eigenvalues after the first component.

Discovery of a high level of internal consistency suggested that a unidimensional scale is likely, but that is not a direct demonstration of such structure. Given the non-normal (bimodal) distribution of item scores and the low resolution of a four-point item scale, a principal components analysis was undertaken, one of the methods least impacted by distribution issues. The correlation matrix indicated all inter-item correlations were above 0.37 and ranged to 0.66, suggesting a promising matrix for factor extraction. Principal component analysis discovered that all item communalities were acceptable, ranging from 0.39 to 0.61. Component extraction revealed that only the first had an eigenvalue exceeding the Kaiser criterion of 1. This component accounted for 53% of the variance (Figure 7). For subsequent components, eigenvalues dropped sharply to below 1 and formed a clear elbow in the scree plot (the Cattell indicator) indicative of a one-component solution, i.e., a unidimensional scale. Item loadings on this first component were all of good magnitude, in a relatively narrow range from 0.62 to 0.78.

## Discussion

The fTORT as constructed works well to form a simple, internally consistent, and unidimensional scale, which is encouraging given the effort taken to select the items. The test was designed to assess recognition of 3D common objects, including amount of specific sensory attribute within an object set (e.g., size of keys). An important feature of the test is use of exploratory procedures; a characteristic of knowledge-driven haptic exploration (Lederman and Klatzky, 1987, 1990). Scale analyses indicate the original 14 item sets devised for object recognition testing form a well-behaved unidimensional scale, with very good internal consistency. The Cronbach alpha was 0.93 and deletion of any item set failed to improve the very good Cronbach alpha. A simple, unweighted addition of the 14 item scores, which is also simple to implement, is supported based on a single component solution with similar loadings across the items. Of interest is the observation that *impaired* performance is dominated by severe error of object recognition (i.e., score of zero), rather than accumulation of simpler one- or two-attribute somatosensory errors. Importantly, we did not observe a skewed distribution within item sets where an item showed *only* 0 scores (i.e., suggesting that item might be too difficult) or *only* 3 scores (i.e., suggesting that item might be too easy). Further, the total score range appears to differentiate individuals over a wide range of object recognition impairment.

The 14 item sets comprising the fTORT were constructed to promote good content and face validity for object recognition, with minimal reliance on language (via use of poster). For stroke and clinician stakeholders, the face validity of the fTORT as a test of the ability to recognize common objects through the sense of touch is argued on the basis that everyday objects are used as test items, that these objects are readily sourced and commonly used, and that real 3D common objects should be used as humans are accurate and efficient in recognizing such objects (Klatzky et al., 1985). The content validity of the test items as representing everyday objects that have key somatosensory features is defended on the basis that all items have been systematically selected from a larger pool of the population of everyday objects (*n* = 100) that have been categorized in relation to the key somatosensory features aligned with haptic exploration of them, as established in the extensive, empirical work of Lederman and Klatzky (1987, 1990, 1993). The test includes item sets that sample each of the known seven somatosensory attributes (Lederman and Klatzky, 1987, 1990, 1993), supporting the content validity of the fTORT as representing all aspects of the construct of haptic object recognition. Further, the test procedure aligns with recognition of those distinctive somatosensory features, i.e., through object pair response choices. Use of visual representation of object features in the response poster, together with opportunity for visual calibration of actual objects above the poster, is defended based on the alignment of visual and tactile object features (Lacey and Sathian, 2014). Finally, the test is designed to minimize impact of confounds such as memory and language.

### The Nature of Haptic Object Recognition Errors

Participants were most often observed to correctly identify the object or not. This pattern was obtained on all item sets with a possible mild deviation only for item 9 where there was some lack of clarity about the upper mode (i.e., exact match and one-attribute sensory error scores were of similar frequency) (Figure 3). The dominance of correct response or complete failure over presence of somatosensory attribute errors was strongest in participants with lower total scores, e.g., 29 or less (62% of cases). Only cases with relatively good total scores (30 or better) showed errors mostly in one or two somatosensory attributes, while correctly identifying the object type.

The work by Lederman and Klatzky (1987, 1990, 2009) identified the somatosensory attributes, and corresponding exploratory procedures, that permit most optimal recognition of common 3D objects by persons without neurological impairment. This information was used both in the selection of a representative range of common objects that are recognized most optimally according to the seven somatosensory attributes, as well as to test the ability to correctly discriminate the amount of the distinctive sensory attribute of similar objects within an object set (i.e., via object pair). It was expected that this higher level of discrimination, in addition to the recognition of object function, would be observed in participants with relatively mild overall impairment. This hypothesis is consistent with the observation that the errors in individuals with mild total score reductions (i.e., scores of 30–39) were predominantly due to somatosensory attribute errors rather than complete failure to recognize the type of object. It suggests that those with relatively few errors may be able to recognize the object function category but miss accurate discrimination of the distinctive somatosensory features of the object or may not be able to distinguish those attributes from other sensory attribute(s) in related objects. The fifth percentile criterion of abnormality in older healthy individuals is 37 out of possible score of 42 (Carey et al., 2006), i.e., within the range where errors are predominantly due to inaccuracy of sensory attribute recognition.

These findings of baseline performance suggest that scoring could be simplified to some degree, i.e., error in both function and sensory attribute versus complete success. However, there is likely value is separating deficits of (1) object function and attribute, (2) one or more sensory attribute errors, and (3) complete correct recognition, based on functional significance for individuals and focus for somatosensory retraining. The ability to detect improvement in specific somatosensory attributes recognized may also permit more sensitive monitoring of change in haptic object recognition over time and in relation to sensory retraining outcomes. A dichotomous score would prevent this insight. Our findings suggest the need for further investigation of test scores over time and their interpretation, given the potential impact on clinical application.

### New Insights Into the Nature of Somatosensory Impairment After Stroke

Our findings suggest new insights into the “sensing brain” and nature of somatosensory impairment after stroke. The observed strong bimodal pattern of scores across all items could reflect an expression of two subsystems. Lederman and Klatzky (1987) describe two haptic subsystems: a “sensory” subsystem that is directed to perception of specific sensory features of spatial layout and structure, and a “motor” subsystem linked with exploratory procedures that enhances the sensory subsystem to efficiently extract and recognize the desired knowledge about objects (e.g., shape) and recognize what the object is (e.g., fork). Lederman and Klatzky highlight that the sensory subsystem may be less than optimal at perceiving specific spatial layout and structure measures when tested in isolation. In comparison, purposive use of exploratory procedures that are optimized to extract knowledge about distinctive object attributes in an interdependent way leads to a very efficient recognition of 3D common objects (Lederman and Klatzky, 1987). The fTORT was designed to assess 3D haptic object recognition using both subsystems, as is typically required in daily activities. The current bimodal distribution of scores could reflect the contribution from these subsystems, when both are working interdependently, or neither are working. For example, a completely correct response on the fTORT may suggest that the subsystems are working successfully together to recognize the desired knowledge about an object (e.g., shape) and what the object is, *as well as* quantity of that distinctive sensory attribute.

The very high proportion of severe errors (score of 0) suggest that stroke survivors have difficulty recognizing object function *and* sensory attributes. The frequency of these severe errors rose steadily with the increase in impairment score and at an earlier point in the impairment scale than expected mathematically. The steady rise in errors observed could be attributed to breakdown of multiple contributing factors and/or to the poor integration of critical capacities. It is also possible that the integrated whole is greater than the sum of its parts, consistent with Lederman and Klatzky’s findings that our haptic system is most efficient when it is enhanced by the motor system and optimal exploratory procedures (Lederman and Klatzky, 1987). A reflection from a stroke survivor captures this interdependence: “…it is like for a blind person their eyes move but they don’t see, it is that, your hand moves but it doesn’t see. And the difference in what you can do when you can feel something as opposed to not feel something just is indescribably different in life” (Turville et al., 2019). Our finding and previous evidence highlights the potential importance of the interdependence of sensory and motor subsystems in optimal object recognition, including the use of exploratory procedures to search for desired knowledge about objects.

Exploratory procedures provide a window into haptic object recognition (Lederman and Klatzky, 1987). They are purposive, knowledge-driven, and may be necessary, sufficient, and/or optimal in the recognition of specific somatosensory attributes. A clinical observation when using the fTORT is that often the stroke survivor does not use the most optimal exploratory movement, even when they have the movement capability. Rather, they frequently employ a global enclosure movement or a non-specific squeezing movement. *Post hoc* review of exploratory procedures recorded in the current study for each of the seven sensory attributes (based on 75% of the sample, as EPs were unclear or unknown for 25%) revealed that the use of the correct EP matched for the sensory attribute in the item set was relatively low, ranging from 31% for part motion/function item sets to 65% for size item sets, mean 50.57% across item sets. In comparison for those who were recorded as having full active movement, when a score of 3 was obtained (i.e., 62% of occasions), the optimal EP was used in 81% of instances, with additional EPs also recorded. For those who required full (*n* = 38) or partial (*n* = 18) guided movement of EPs from the assessor during testing (total *n* = 56), 56% reported a score of zero, whereas 28% achieved a score of 3 (indicating a score of 3 is still possible with guided exploratory procedures).

### Dimensionality of the fTORT, Distribution of Performance Errors, and Internal Consistency of Test Items

The fTORT was designed to assess recognition of 3D common objects (involving clusters of features relating to object function) and to discriminate/recognize the amount of a specific sensory attribute within an object set (e.g., different sizes of keys). The total score involves simple addition of the 14 item scores, based on evidence of a unidimensional scale with similar loadings across test items. The principal component result (Figure 7) provides clear evidence of a one-component solution, i.e., a unidimensional scale, with all item loadings of good magnitude and in a relatively narrow range from 0.62 to 0.78. The high loading across all 14 items suggests commonality and meaning. The items are common in that they sample recognition of 3D common objects through the sense of touch (vision occluded), and are closely aligned with the objects and construct of haptic object recognition empirically tested and validated by Lederman and Klatzky (1987, 1990, 1993). Although objects were also selected to differ in sensory attributes optimal for recognition, all objects were everyday objects, requiring attentive exploration, and appearing to need a combination of haptic tactile and proprioceptive input to be correctly sensed and recognized. The spread of items across the seven diagnostic attributes of sensation support previous evidence that each of these attributes contributes to haptic object recognition and may suggest a dependence on multisensory input and interpretation. The spread of error scores across the stroke sample also suggests that the impairment after brain injury is sufficiently distributed, such that patterns of error across specific sensory attributes did not emerge to create a multicomponent structure.

The spread of total scores across the full range of possible scores, from normal performance to most severe impairment, suggests that the 14-item measure worked well, at least for the current sample. The wide spread of scores, shown in Figure 6, is not unexpected given the high variability in stroke severity and lesion location, and the complex processing that is thought to occur in haptic object recognition. The spread suggests presence of a range in severity of impairment, consistent with existing literature (Carey, 1995; Connell et al., 2008; Tyson et al., 2008; Carey and Matyas, 2011; Kessner et al., 2016). The slight increase in frequency at scores of 40 and 41 is indicative of unimpaired performance relative to age-matched healthy controls (Carey et al., 2006). The pooled cohort represents stroke survivors who were screened clinically for presence of somatosensory impairment, are able to follow at least two-stage commands, are able to participate in rehabilitation, and do not have neglect. There were more men than women and the mean age is lower than the general population of stroke survivors (Feigin et al., 2003), although the burden of stroke in people younger than 65 years has increased over the last few decades (Katan and Luft, 2018). Nevertheless, the sample was relatively heterogeneous, including those with cortical and/or subcortical lesions, right or left hemisphere lesions, and ischemic or hemorrhagic stroke. It included people who had either the dominant (56%) or non-dominant (44%) hand affected, and were at varying times post-stroke, ranging from 3 to 129 weeks post-stroke. Thus, they may be considered relatively representative of the population of stroke survivors who present for rehabilitation (Carey and Matyas, 2011).

Object sets included were carefully selected to sample the full range of object sensory attributes (Lederman and Klatzky, 1987) and a wide range of 3D objects commonly encountered and previously categorized according to object function and corresponding optimal EP (Lederman and Klatzky, 1990). Despite this range, item means and SDs did not differ markedly, suggesting a limited range of item difficulty and discriminability potential. This finding suggests that there is no difficulty hierarchy evident across items. Further, the items that contributed low scores varied for people with the same total score, and higher total scores were obtained from high scoring items across a variety of items. The representativeness of sampled individuals and test objects increases confidence that errors across an increasing number of item sets indicates more severe impairment. The wide spread of scores also suggests the potential for future determination of levels of impairment severity across the scale. Presence of errors in object function and sensory attribute (score of 0) is suggestive of an impairment, even for relatively mild total impairment scores. In addition to the total impairment score, therapists can gain insight into the nature of impairment – i.e., errors that include recognition of object function and errors relating to specific sensory attributes (or modalities) for the individual tested.

### Knowledge-Driven Haptic Exploration and the fTORT

The fTORT provides an assessment of tactile object recognition that aligns with how the haptic object recognition system works in recognizing common everyday objects. It requires recognition of object function and discrimination of specific somatosensory attributes, potentially requiring both sensory and motor haptic subsystems (Lederman and Klatzky, 1987). Extraction of object features and sensory attributes is prompted by the object sets visually displayed on the poster, and hence is set up for knowledge-driven exploration. The test aims to capture the interdependence of sensory object attributes and the exploratory procedures used to extract them. It uses real 3D objects and sensory attributes that are typically recognized using the matched optimal exploratory procedure.

An important part of testing is the observation of exploratory procedures actually used. Exploratory procedures are recorded by the therapist, with the most optimal exploratory procedure identified on the testing form to prompt observation and recording. Although these observations are not used in scoring, they provide information on how the person explores the object and this can be used in therapy. In cases where movement is limited, the therapist uses standardized guided movement of the most optimal exploratory procedure, matched for a given object set to make sure an adequate stimulus is presented. Although time taken to recognize objects haptically is important for everyday function, the response time may be impacted by impairment in motor control after stroke. Time was recorded to monitor the expected efficiency of haptic object recognition, as observed in adults without stroke or movement deficits. It may be of value when testing those with only mild deficits. We recommend recording the time taken, but did not limit the time nor penalize for longer time taken in the fTORT, especially as in some instances guided movement was required.

### Testing Procedures and Implications

The fTORT was designed to assess rapid recognition of objects through the sense of touch. During testing, participants were instructed that they would be timed, but were given a relatively unrestricted time to explore the object and its distinctive somatosensory attribute (prompted by the response poster). Participants were encouraged to give a response within 60 s, although some participants took more than 30 or 60 s to discriminate the object and distinctive somatosensory attribute. In other tests, a time of greater than 30 s may be interpreted as an error (Carey, 1995). In the fTORT, response was timed but scoring was based on response errors. The additional time allowed may have permitted some to achieve the maximum correct score of 3 only after extensive and deliberate searching and recognition.

The fTORT requires the person to attend to object features during exploration (active or guided) and then to nominate their response using the response poster. Importantly, the object poster is in full view throughout object exploration (minimizing memory confounds) and the participant is reminded that they are to point to or name the object (or object number) that most closely matches the object that they are feeling out of view. A possible explanation for the high proportion of severe error scores could be a lack of understanding of test instruction and/or impaired attention and cognition. However, this is an unlikely explanation as participants were screened for cognition, some items were correctly matched during testing, and most participants showed scores within the normal range, or at least significantly better, for the “unaffected” hand. The standard protocol permits reminders of test protocol by the assessor if required.

### Implications for Assessing Haptic Object Recognition and Clinical Practice

Our findings have implication not only in relation to better understanding the nature of haptic object recognition errors observed in people who experience somatosensory impairment after stroke but also the type of measurement tools used to assess this capacity. To date, quantitative measures have tended to focus on a single attribute alone, such as shape (Rosen and Lundborg, 1998; Kalisch et al., 2012), rather than discrimination and integration of multiple attributes in the context of 3D real objects. In comparison, clinical testing has involved recognition of non-standard everyday objects without knowing whether the range of somatosensory object attributes are being adequately sampled nor whether a person can discriminate differences in distinctive somatosensory attributes (Carey, 1995). It is argued that the fTORT represents one step forward in capturing haptic object recognition of real 3D objects after stroke, with quantification of the extent and nature of object recognition errors. The fTORT assessment has also been adapted and tested for use with children with cerebral palsy (Taylor et al., 2018). The adapted test demonstrated preliminary construct validity and was positively associated with an upper limb activity measure (Taylor et al., 2018).

### Future Directions

Future studies should establish age-matched normative standards andthe discriminative validity of the test with larger samples, beyond the early preliminary data reported to date (Carey et al., 2006), as well as retest and inter-assessor reliability. In addition, empirical investigation of the criterion validity of the fTORT as a measure that relates to and/or predicts recognition and functional use of such objects in real-world contexts by stroke survivors would be of benefit to support clinical use. It would help to establish concurrent validity for outcomes measured at the same time and/or predictive validity for future outcomes. One potential limitation of use of the test across different cultures and over time relates to the familiarity of the common objects included as items in the test. For example, a fork is likely to be less familiar in Asian populations, whereas a clothes peg may not be so commonly used in the future. The potential exists to adapt some objects to different cultures.

The fTORT includes 14 item sets to assess haptic object recognition, sampling the seven attributes of sensation twice. Sampling each attribute twice was the minimal testing burden possible to investigate if multiple sampling of an attribute is needed, given likely complexity of information processing demanded by real-world objects. Our findings support initial selection of 14 items on the basis that each object attribute is only tested twice, correlations for attribute pairs are not overly high (ranging from 0.37 to 0.64), and longer tests are theoretically more reliable than shorter tests, unless items are highly correlated. However, the ultimate decision on length of a test is a compromise between opposing test design objectives: brevity that saves time and minimizes fatigue versus higher reliability. At this stage of development and testing, inclusion of two item sets for each attribute permitted initial investigation of whether a specific attribute (e.g., shape) is consistently impaired and could inform selection of item sets for future investigations. However, redundancy among items was not assessed in detail within this work. Future investigations may reveal the feasibility of shorter test duration and the best item combination, which would be of value to support the clinical utility of the tool.

Further investigation of the relationship between item scores and exploratory procedures employed would be of value to help unravel the nature of the disruption to knowledge-driven haptic recognition after stroke. Investigation of type and severity of response error over time may also be of value to better understand features of haptic object recognition that may change over time and/or be impacted by sensory rehabilitation. The impact of factors such as side of lesion and brain networks affected by the stroke (including somatosensory, motor, and multimodal processing hubs) may also help to better understand the nature of the impairment and the role of connected regions and networks that could contribute to recovery and rehabilitation. Evidence of how the somatosensory and motor systems can work together within a knowledge-driven framework suggests important pathways for development of interventions that directly use this knowledge. The SENSe (*S*tudy of the *E*ffectiveness of *N*eurorehabilitation on *Se*nsation) approach (Carey et al., 2011) is one such therapy that helps stroke survivors regain a sense of touch and better recognize the function and sensory attributes of real objects through a perceptual learning approach coupled with principles of neuroscience, specific training modules (Carey, 2012), and carefully designed and graded therapeutic equipment. The success of this approach has been demonstrated in a randomized controlled intervention study (Carey et al., 2011). In line with this special issue on the sensing brain, the potential value of combining training of sensation and movement (Gopaul et al., 2019) in an integrated manner for goal-oriented action is also highlighted.

## Conclusion

In conclusion, the fTORT functions well as a unidimensional scale, supporting simple addition of the 14 item scores on the fTORT. The excellent internal consistency of items supports assessment of haptic object recognition using the item sets selected. Therapists can use the fTORT to quantify impaired tactile object recognition in people with stroke. New insights into the nature of somatosensory impairment after stroke are revealed.

## Data Availability Statement

The datasets generated for this study are available on reasonable request to the corresponding author.

## Ethics Statement

All participants gave voluntary informed consent and procedures were approved by Human Ethics committees of participating hospitals and La Trobe University, Australia.

## Author Contributions

LC wrote the article, developed the measurement tool, conceived and conducted the studies, designed the pooled data study, obtained ethics, and contributed to data analysis and interpretation of results. YM-Y extracted and pooled data from existing studies, assisted with data collection, and contributed to data analysis and article drafting and editing for important intellectual content. TM contributed to the conception of the study, led and conducted the data analysis, contributed to drafting and editing of the article for important intellectual content, and had a major input to interpretation of results. All authors contributed to the article and approved the submitted version.

## Conflict of Interest

LC is the originator of and led the development of the functional Tactile Object Recognition Test. The test is now available for purchase as part of an evidence-based assessment and training unit from a not-for-profit organization (The Florey Institute of Neuroscience and Mental Health). The remaining authors declare that the research was conducted in the absence of any commercial or financial relationships that could be construed as a potential conflict of interest.
